# Hyaline and Cystic Degeneration of Uterine Leiomyomas: CT and MR Imaging with Histopathological Sample Analyses

**DOI:** 10.3390/diagnostics13203230

**Published:** 2023-10-17

**Authors:** Camilo G. Sotomayor, Carla Parra, Maximiliano Miranda, Juan Salvador Casas, Gonzalo Cárdenas, Álvaro Sanhueza, Francisca Araya, Iván Gallegos, Sebastián Yévenes

**Affiliations:** 1 Abdominal and Pelvis Unit, Radiology Department, Clinical Hospital University of Chile, Faculty of Medicine, University of Chile, Independencia, Santiago 8380453, Chile; 2 Anatomy and Developmental Biology Program, Institute of Biomedical Sciences, Faculty of Medicine, University of Chile, Independencia, Santiago 8380453, Chile; 3 Pathology Department, Clinical Hospital University of Chile, Faculty of Medicine, University of Chile, Independencia, Santiago 8380453, Chile

**Keywords:** uterine leiomyoma, cyst degeneration, hyaline degeneration, computed tomography, magnetic resonance imaging

## Abstract

Leiomyomas are the most common solid benign uterine neoplasms; they are usually asymptomatic and are identified incidentally. Yet, responsive to stimulation by estrogens, leiomyomas may expand, potentially outgrowing their blood supply to undergo hemorrhage, fibrosis, calcification, and atrophy. These pathologic mechanisms commonly lead to leiomyomas degeneration, i.e., red, hyaline, cystic, or myxoid. Magnetic resonance (MR) imaging is the most accurate imaging technique for the characterization of leiomyomas. In cases of degeneration, variable features on T2-weighted and contrast-enhanced images can be found. With no recent radiologic pathologic correlation literature available on this matter, herewith, we provide computed tomography (CT)/MR imaging along with histopathological specimens of two young women who were diagnosed with hyaline or hyaline and cyst degeneration of uterine leiomyomas at our university hospital. We report on the imaging features of uterine leiomyomas using CT and MR imaging and discuss the available literature on imaging signs that may be suggestive of hyaline or cyst degeneration using either of the imaging examination methods.

**Figure 1:**
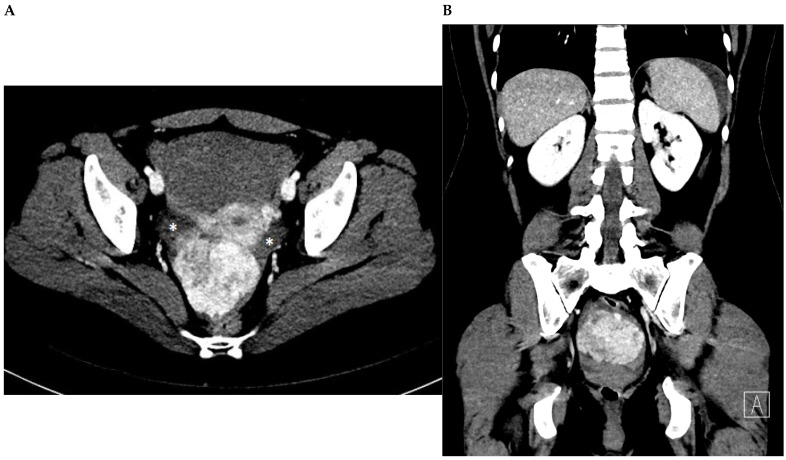
A woman without comorbidities, a non-oral contraceptive user, and without sexual activity for 2 years, presented to the emergency department with abrupt onset 6 h before featuring non-characteristic lower abdominal pain of mild intensity, i.e., visual analog scale (VAS) 6/10. The pain increased steadily in severity to VAS of 10/10. The patient denied history of fever, gastrointestinal or urinary complains, or genital bleeding or discharge. Upon physical examination, the abdominal area was found to be painful without peritoneal irritation signs. The patient underwent computed tomography examination (Figure 1) and surgery with total myomectomy and specimen histopathological analyses (Figure 2). Herein, Figure 1 shows a subserosal-pedunculated leiomyoma with hyaline degeneration demonstrated by biopsy (Figure 2) in a 32-year-old woman. (**A**) Axial and (**B**) coronal contrast-enhanced computed tomography images reveal a normal sized uterus with thin endometrium. From the posterior aspect of the corpus, there is a well-circumscribed subserosal-pedunculated mass attached to the serosa by a narrow stalk (International Federation of Gynecology and Obstetrics [1]; FIGO stage 7). It measures approximately 72 × 56 mm on the axial plane and shows heterogenous enhancement. There are small, focal, non-enhancing areas of cystic appearance, which may be suggestive of hyaline degeneration or necrosis (up to 30% of histological distribution of degeneration area). Dense fluid is also observed surrounding the uterus, which is consistent with hemoperitoneum (*) and suggests acute complication of the mass.

**Figure 2:**
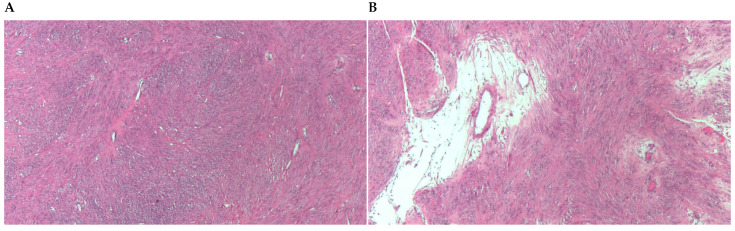
(**A**) Leiomyoma with architecture of perpendicularly intertwined bundles, of medium cellularity and low cytological atypia. (**B**) Approach to the focus of hydropic degeneration, with extracellular edema and separation of the tumor cells.

**Figure 3:**
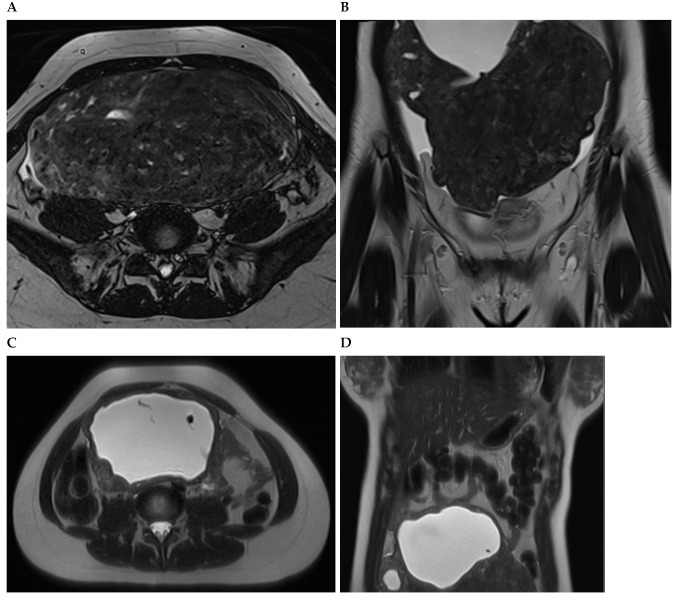

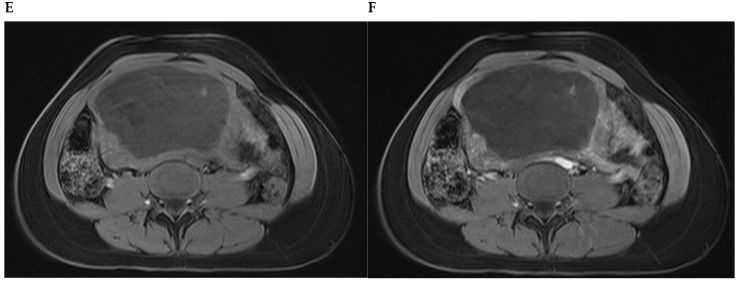
A woman with non-oral contraceptive user, presented to the outpatient clinic requesting a second opinion concerning a 240 mm sized uterine leiomyioma found by ultrasound at a different healthcare center. A computed tomography showed a uterine multinodular lesion with signs of degeneration. The patient received magnetic resonance imaging examination (Figure 3) and surgery with total myomectomy and specimen histopathological analyses (Figure 4). Herein, Figure 3 shows a subserosal-pedunculated leiomyoma with hyaline and cystic degeneration in a 29-year-old woman. (**A**) Axial and (**B**) coronal T2-weighted (T2) Dixon magnetic resonance imaging (MRI) showed a normal-sized uterus with axial organ rotation by 180° in an anticlockwise direction. From the right cornual region of the uterus, there was a low signal intensity (T2) subserosal-pedunculated mass (FIGO 7). This well-circumscribed, giant abdominopelvic mass measured approximately 173 × 84 × 174 mm and presented signs of cystic degeneration on its most cranial portion by demonstrating an internal, round, well-defined area with homogeneous fluid-like high signal intensity (T2), best represented on (**C**) axial and (**D**) coronal T2 half-Fourier acquisition single-shot turbo spin-echo MRI. It also demonstrated (**E**) low-signal intensity on non-contrast axial T1-weighted (T1) Dixon MRI, (**F**) without enhancement on arterial axial T1 Dixon MRI, further suggesting cystic degeneration (up to 80% of histological distribution of degeneration area) of this giant leiomyoma.

**Figure 4:**
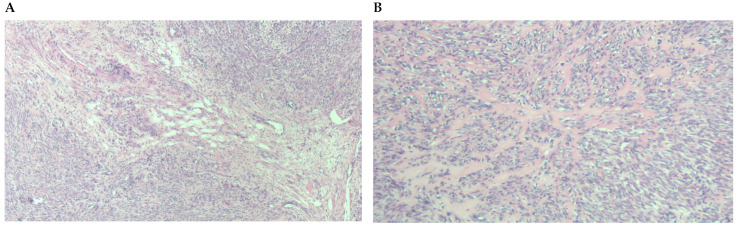
(**A**) Tumor with small foci of hydropic degeneration maintaining benign characteristics. (**B**) Close-up of a focus of hyaline degeneration with collagen interspersed with tumor cells.

We provide two cases of young women with hyaline and cystic degeneration of uterine leiomyomas, hereby demonstrated by histopathological sample analyses, which illustrate features and imaging signs that may suggest hyaline or cyst degeneration upon CT and MR imaging. It should be noted, however, that computed tomography may not invariably allow for the differentiation of degeneration types [2]. The European Society of Urogenital Radiology (ESUR) Guidelines report that 80% of European centers do not see any role for computed tomography when assessing uterine leiomyomas [3]. Yet, pain occurs in approximately 30% of women with uterine leiomyomas, particularly as result of acute degeneration [4]. Thus, in the acute setting of a woman presenting to the emergency department with lower abdominal pain, CT findings suggestive of uterine leiomyoma degeneration are clinically relevant as it may be the underlying cause of pain, e.g., lower abdominal pain steadily increased to VAS of 10/10 in the patient from case 1. The most important point of imaging of uterine leiomyomas is, indeed, detecting malignant transformation or leiomyosarcoma, and recent literature suggests that MRI features may aid in the differentiation between leiomyoma and leiomyosarcoma. Yet, imaging findings of leiomyoma degeneration in the era of advanced imaging techniques remain scarcely described, even in most recent publications [5]. Hyaline degeneration is the most common type of degeneration, yet its differentiation from a non-degenerated leiomyoma using computed tomography is virtually not possible. Three-phase dynamic MRI and diffusion weighted imaging with very small b-factors has been proposed for differentiating completely hyalinized leiomyomas from ordinary leiomyomas [6], further underscoring the limited role of computed tomography when assessing uterine leiomyomas. Thereafter, hyaline degeneration tends to evolve into liquefication. In cases of degeneration of a giant leiomyoma (e.g., patient from case 2), hyaline degeneration (areas with heterogenous low signal intensity on T2 images) usually evolves into a large cystic cavity (area with homogeneous fluid-like high signal intensity on T2 images), potentially simulating pregnancy or ovarian cyst. These and other causes of uterine and adnexal masses in the female pelvis are relevant differential diagnoses in the appropriate clinical and imaging setting [7]. Considering recent images on the radio-pathological correlation of uterine leiomyomas degeneration, we plea for further and deeper up-to-date articles on this topic. We acknowledge that macroscopic photographs or loupe photos of cut surfaces of myomectomy tumors were not available, which would have also allowed comparison of imaging and pathologic findings. With novel imaging techniques, currently, there is a relative scarcity of updated literature providing a systematic review of CT and particularly MR imaging features of uterine leiomyomas with different types of degeneration, pointing towards the need for revised radio-pathological correlation studies in the field [2,6,7,8].

## Data Availability

Data available upon request due to privacy restrictions.
